# Purification by Fire

**Published:** 1900-11-17

**Authors:** 


### PURIFICATION BY FIRE.

Dealing with the same subject from a rather different point of view, Major Bissell, M.D., of the United States Army, writing in the New York *Medical Record*, describes what has lately been done in the direction of incinerating excreta. The apparatus which has been used this year at the camp of instruction of the National Guards of New York consists of a waggon containing eight "incinerators," and two of these waggons were found sufficient to deal with the excreta of over 500 men during their term of field service. The incinerator was constructed in such a manner that the excrement was first evaporated and then burned without ever having been removed from the iron pan in which it had been deposited, and the arrangement was such that the gases arising from the process of incineration were passed through the firebox of the furnace, with the object of preventing smell. This result, however, does not seem to have been completely attained, but so far as the sterilisation of the material is concerned the apparatus seems to have been quite successful. The arrangement, however, seems to us to be heavy and cumbrous, and the use of so many separate fires as are provided is certainly a wasteful and unscientific proceeding. Æsthetically, no doubt, there are many reasons for preferring incineration as a means of disinfecting excreta, but after all there can be no doubt that, in comparison with boiling, it is a wasteful process, the heat required to raise the material to the boiling temperature being a trifle compared with that required to evaporate it to dryness and cause incineration. If burning is to be aimed at, some form of furnace in which the heat contributed by the combustion of one portion should be used to assist the burning of the next would seem essential. Where, however, as is the case with armies in the field, every pound of fuel is of importance, it will probably be found most economical to be content with the lowest heat by which sterilisation can be assured—namely, that of boiling.

